# A Post-Processing Multipath/NLoS Bias Estimation Method Based on DBSCAN

**DOI:** 10.3390/s24082611

**Published:** 2024-04-19

**Authors:** Yihan Guo, Simone Zocca, Paolo Dabove, Fabio Dovis

**Affiliations:** 1 Department of Electronics and Telecommunications, Politecnico di Torino, 10129 Turin, Italy; simone.zocca@polito.it (S.Z.); fabio.dovis@polito.it (F.D.); 2 Department of Environment, Land and Infrastructure Engineering, Politecnico di Torino, 10129 Turin, Italy; paolo.dabove@polito.it

**Keywords:** multipath, non-line-of-sight, pseudorange bias, clustering algorithm

## Abstract

Positioning based on Global Navigation Satellite Systems (GNSSs) in urban environments always suffers from multipath and Non-Line-of-Sight (NLoS) effects. In such conditions, the GNSS pseudorange measurements can be affected by biases disrupting the GNSS-based applications. Many efforts have been devoted to detecting and mitigating the effects of multipath/NLoS, but the identification and classification of such events are still challenging. This research proposes a method for the post-processing estimation of pseudorange biases resulting from multipath/NLoS effects. Providing estimated pseudorange biases due to multipath/NLoS effects serves two main purposes. Firstly, machine learning-based techniques can leverage accurately estimated pseudorange biases as training data to detect and mitigate multipath/NLoS effects. Secondly, these accurately estimated pseudorange biases can serve as a benchmark for evaluating the effectiveness of the methods proposed to detect multipath/NLoS effects. The estimation is achieved by extracting the multipath/NLoS biases from pseudoranges using a clustering algorithm named Density-Based Spatial Clustering of Applications with Noise (DBSCAN). The performance is demonstrated using two real-world data collections in multipath/NLoS scenarios for both static and dynamic conditions. Since there is no ground truth for the pseudorange biases due to the multipath/NLoS scenarios, the proposed method is validated based on the positioning performance. Positioning solutions are computed by subtracting the estimated biases from the raw pseudoranges and comparing them to the ground truth.

## 1. Introduction

Multipath and Non-Line-of-Sight (NLoS) aspects are the most detrimental effects for Global Navigation Satellite Systems (GNSSs) in urban scenarios, affecting the large plethora of emerging applications being developed and expected to work in such an environment, such as autonomous vehicles, smart wearables, etc. [1]. Multipath interference, as the name implies, happens when a signal is received through multiple paths. This can include the direct path and one or more reflected paths, or it may involve multiple reflected paths. NLoS reception occurs when the direct path from the transmitter to the receiver is obstructed and the signals are received only through a reflected path [2].

The presence of reflected GNSS signals in the case of multipath/NLoS effects causes biases ranging from several to thousands of meters in pseudorange measurements [3]. Unlike the tropospheric and ionospheric delay contribution and relativistic effect, whose physical models allow for proper correction, mitigating the impact of multipath/NLoS effects on pseudorange measurements is challenging due to their complex physical models.

As multipath/NLoS effects pose critical threats to the widespread use of GNSS, numerous efforts have been devoted to detecting and mitigating the pseudorange biases caused by such propagation conditions. Most of the research efforts can be categorized into three groups based on the specific stage of the user receiver at which detection and/or mitigation are applied:

The first group of solutions includes antenna-based techniques. In scenarios involving multipath/NLoS effects, the reflected GNSS signals may undergo changes in polarization and reception angles compared to the direct signals. To mitigate this issue, researchers developed advanced antenna designs, including choke rings, dual-polarized antennas, and array antennas, to minimize the reception of reflected signals [2,4,5].

The second family of multipath/NLoS detection and mitigation techniques relies on signal processing. Multipath occurs when the signals reflected from surfaces reach the receiver along with the direct signals. In GNSS baseband signal processing, the shift in auto-correlation functions caused by reflected signals can overlap with the auto-correlation function of the direct signal. As a result, the computed auto-correlation function in the Delay-Lock Loop (DLL) will be distorted due to this overlapping. To address this, various advanced signal tracking techniques have been proposed for identifying and mitigating multipath interference [6,7,8,9].

The third group of techniques operates within the positioning unit to detect and mitigate multipath/NLoS interference. Pseudorange biases induced by multipath/NLoS effects pose a threat to the consistency between the pseudorange measurements and the corresponding navigation solutions. Some approaches utilize the chi-squared test for detecting and mitigating the impact of multipath/NLoS effects [10]. Additionally, 3D city models and cameras are employed to establish the propagation model of reflected signals [11,12]. Furthermore, researchers explore the potential of machine learning algorithms to establish relationships between multipath/NLoS effects and other indicators, such as satellite elevations/azimuths and the carrier-to-noise-density ratio ($C/N_{0}$) [13,14,15].

In an effort to contribute to the development of methodologies mitigating multipath/NLoS effects, this research proposes a post-processing solution for estimating the pseudorange biases caused by these phenomena. In recent years, new research approaches have considered machine learning-based algorithms for detecting and mitigating multipath/NLoS effects. By extracting certain features in the positioning unit, ref. [11] proposed a classifier based on supervised machine learning to categorize GNSS pseudorange measurements into three types: clean, multipath, and NLoS. Moreover, ref. [16] adopted an unsupervised method to identify satellite signals with multipath using carrier phase, pseudorange, and carrier-to-noise ratio measurements. Additionally, ref. [17] formulated multipath modeling as a regression task, fitting the multipath errors with respect to the azimuth and elevation in the spatial domain. To implement multipath detection and mitigation, these studies utilized various features derived from GNSS raw data as well as additional information. Accurate values of pseudorange biases due to multipath/NLoS effects are pieces of information essential for these machine learning approaches to train the model, and then they are used to detect and mitigate multipath/NLoS effects. Therefore, the primary objective of this research is to provide high-precision pseudorange bias values caused by multipath/NLoS to empower machine learning-based algorithms. Furthermore, these accurately estimated pseudorange biases can serve as a standard for assessing the performance of the methods proposed for detecting and mitigating multipath/NLoS effects.

To compute the accurate pseudorange biases caused by multipath/NLoS effects, this research leverages precise corrections and models for GNSS and employs a clustering algorithm, namely Density-Based Spatial Clustering of Applications with Noise (DBSCAN). The main novel contributions of this work can be summarized as follows:

1. Building upon the theoretical analysis of the pseudorange function, this research defines a leftover term containing the pseudorange biases caused by multipath/NLoS effects.
2. This research derives two probability distributions for the defined leftover term, which motivates the utilization of a clustering algorithm to estimate multipath/NLoS effects.
3. By utilizing a clustering algorithm, specifically DBSCAN, to isolate the other components in the defined leftover term, a procedure is proposed to estimate the values of multipath/NLoS biases if multipath/NLoS effects are present.

The paper is organized as follows: in Section 2, the mathematical foundations for the pseudorange function are established, and the definition of the leftover term is provided. Section 3 introduces the proposed method for extracting pseudorange biases caused by multipath/NLoS effects from the leftover term using a clustering algorithm. Subsequently, Section 4 outlines the details of the real-world experiments under both static and dynamic scenarios to validate the effectiveness of the proposed method. Finally, Section 5 draws the conclusions regarding this work.

## 2. Definition and Computation of the Leftover Term

This section recalls the derivation of the pseudorange observation equation, as outlined in [18], in order to provide the definition of the leftover term containing the pseudorange biases due to multipath/NLoS effects, along with other receiver-related error components.

The GNSS receiver generates pseudorange measurements by multiplying the signal travel time from the satellite to the receiver with the speed of light. It is noted that the superscript *s* is used to represent the pseudorandom noise (PRN) code of a specific GNSS satellite, and the subscript *r* is used to represent the GNSS receiver. The pseudorange measurement function is written as

(1) $$\begin{array}{c} p_{r}^{s}\left(t\right)=c\left(d_{r}+dt_{r}\left(t\right)\right)+∥r^{s}\left(t\right)+r_{r}\left(t\right)∥\\ +c\left(d^{s}-dt^{s}\left(t\right)+\delta t_{\mathrm{stc}}^{s,\mathrm{rel}}\left(t\right)-\delta t_{\mathrm{clk}}^{s,\mathrm{rel}}\left(t\right)\right)\\ \;\;\;\;\;\;\;\;\;\;\;\;\;\;\;\;\;\;\;\;\;\;\;\;\;\;\;\;\;\;\;\;\;\;\;\;\;\;\;\;\;\;\;\;\;\;\;\;\;\;\;\;\;\;\;\;\;\;\;\;\;\;\;\;\;\;\;\;\;\;\;\;\;\;\;\;\;\;\;\;\;+\xi_{r}^{s}\left(t\right)+I^{s}\left(t\right)+T^{s}\left(t\right)+F^{s}\left(t\right)+e_{r}^{s}\left(t\right) \end{array}$$

where

- $p_{r}^{s}\left(t\right)$ is the pseudorange measurement obtained from the GNSS receiver for satellite *s* at time *t*;
- *c* is the speed of light in vacuum;
- $d_{r}$ is the signal instrumental delay of the receiver;
- $dt_{r}\left(t\right)$ is the clock bias of the receiver at time *t*;
- $r^{s}\left(t\right)$ is the mass center position of the satellite under Earth Centered Earth Fixed (ECEF) frames at time *t*;
- $r_{r}\left(t\right)$ is the antenna reference point position of the receiver under ECEF frames at time *t*;
- $d^{s}$ is the signal instrumental delay of the satellite;
- $dt^{s}\left(t\right)$ is clock bias of the satellite at time *t*;
- $\delta t_{\mathrm{stc}}^{s,\mathrm{rel}}\left(t\right)$ is the delay caused by space–time curvature of the relativistic effect at time *t*;
- $\delta t_{\mathrm{clk}}^{s,\mathrm{rel}}\left(t\right)$ is satellite clock bias caused by the relativistic effect at time *t*;
- $\xi_{r}^{s}\left(t\right)$ is antenna phase center corrections for both transmitting and receiving antennas at time *t*;
- $I^{s}\left(t\right)$ is the error contribution of the pseudorange measurement due to the ionospheric delay, expressed in meters at time *t*;
- $T^{s}\left(t\right)$ is the error contribution of the pseudorange measurement due to the tropospheric delay, expressed in meters at time *t*;
- $F^{s}\left(t\right)$ is the error contribution of the pseudorange measurement due to the multipath/NLoS interference at time *t*, expressed in meters;
- $e_{r}^{s}\left(t\right)$ is the error contribution of the pseudorange measurement due to the receiver noise at time *t*, expressed in meters.

The Sagnac effect caused by Earth’s rotation should also be compensated for $p_{r}^{s}\left(t\right)$ according to [18] (Chapter 19.1). By adjusting both sides of (1), we can obtain

(2) $$\begin{array}{c} F^{s}\left(t\right)+e_{r}^{s}\left(t\right)+c\left(d_{r}+dt_{r}\left(t\right)\right)\\ \;\;\;\;\;\;\;\;\;\;\;\;\;\;\;\;\;\;\;\;\;\;\;\;\;\;\;\;\;\;\;\;\;\;=p_{r}^{s}\left(t\right)-∥r^{s}\left(t\right)-r_{r}\left(t\right)∥-\xi_{r}^{s}\left(t\right)-I^{s}\left(t\right)-T^{s}\left(t\right)\\ \;\;\;\;\;\;\;\;\;\;\;\;\;\;\;\;\;\;\;\;\;\;\;\;\;\;\;\;\;\;\;\;\;\;\;\;\;\;\;\;\;\;\;\;\;\;\;\;\;\;\;\;\;\;\;\;\;\;\;\;\;\;\;\;\;\;\;\;\;\;\;\;\;\;\;\;\;\;\;-c\left(d^{s}-dt^{s}\left(t\right)+\delta t_{\mathrm{stc}}^{s,\mathrm{rel}}\left(t\right)-\delta t_{\mathrm{clk}}^{s,\mathrm{rel}}\left(t\right)\right) \end{array}$$

Thanks to the advancements in the development of physical models and corrections offered by International GNSS Service (IGS), all the terms on the right-hand side of (2) can be computed in a post-processing manner, with relatively high accuracy, as outlined in Table 1. The left-hand side of (2) is designated as the leftover term $L^{s}\left(t\right)$ of a pseudorange measurement:

(3) $$\begin{array}{cc} L^{s}\left(t\right) & =F^{s}\left(t\right)+e_{r}^{s}\left(t\right)+c\left(d_{r}+dt_{r}\left(t\right)\right)\\ \; & =F^{s}\left(t\right)+e_{r}^{s}\left(t\right)+c\;\cdot \;dt_{\mathrm{rcv}} \end{array}$$

Since $d_{r}$ and $dt_{r}\left(t\right)$ are produced by the GNSS receiver itself, $d_{r}$ remains constant under a specific epoch *t* for different satellites. Furthermore, the same $dt_{r}\left(t\right)$ is also shared for every satellite under a certain epoch *t*. Therefore, these two terms can be combined as a single term, which is denoted as $dt_{\mathrm{rcv}}$.

## 3. Multipath/NLoS Bias Estimation Using a Clustering Algorithm

This section will elaborate on the method proposed by this research to estimate the pseudorange biases caused by multipath/NLoS effects. First, two probability distributions of leftover terms are generated, distinguishing between conditions with and without multipath/NLoS effects. These distinct distributions motivate the use of clustering algorithms to classify the leftover terms affected by multipath/NLoS effects from those that are not. Subsequently, we provide a detailed introduction to a density-based clustering algorithm, namely DBSCAN, and explain its suitability for this task of identifying multipath/NLoS events. Finally, this section illustrates the proposed procedure of utilizing DBSCAN for multipath/NLoS estimation.

### 3.1. Statistical Characterization of the Leftover Terms

Analyzing $L^{s}\left(t\right)$ as in (3), different components with specific features can be recognized:

1. The error contribution of the pseudorange measurement due to the multipath/NLoS interference $F^{s}\left(t\right)$ is usually different from one satellite to another. Furthermore, $F^{s}\left(t\right)$ is zero if a satellite is free from both multipath and NLoS interference.
2. The receiver noise $e_{r}^{s}\left(t\right)$ is commonly characterized by a Gaussian distribution with a zero mean and constant variance $\sigma^{2}$ under multipath/NLoS-free conditions [19]. However, when multipath/NLoS effects occur, the receiver noise still follows a Gaussian distribution with a zero mean and a different variance $\sigma_{F}^{2}$ [20].
3. The user clock bias term $dt_{\mathrm{rcv}}$ keeps the same value for every satellite for a certain epoch.

In both conditions, with and without multipath/NLoS effects, $e_{r}^{s}\left(t\right)$ is relatively small compared to $F^{s}\left(t\right)$. Typically, in the absence of multipath/NLoS effects, the range error due to receiver noise is in the order of ±1 m for a geodetic-quality receiver and antenna [21]. According to [20], the variance $\sigma_{F}^{2}$ increases by approximately two times compared to $\sigma^{2}$ when multipath/NLoS effects occur. However, the ranging bias due to multipath can reach up to about 70 m for GPS L1 C/A signals with one-chip early-to-late spacing [22]. Moreover, NLoS conditions may induce biases in pseudorange measurements spanning several kilometers [3].

Given that $dt_{\mathrm{rcv}}$ and $F^{s}\left(t\right)$ are constant for a given epoch, the leftover term $L^{s}\left(t\right)$ for a specific satellite can be statistically modeled as Gaussian random variables in both conditions, whether multipath/NLoS effects occur or not.

(4) $$L^{s}\left(t\right)∼\left\{\begin{array}{cc} N\left(c\;\cdot \;dt_{\mathrm{rcv}},\sigma^{2}\right) & \mathrm{without}\;\mathrm{multipath}/\mathrm{NLoS}\\ N\left(c\;\cdot \;dt_{\mathrm{rcv}}+F^{s}\left(t\right),\sigma_{F}^{2}\right) & \mathrm{with}\;\mathrm{multipath}/\mathrm{NLoS} \end{array}\right.$$

The proposed method in this research leverages the consistency checking principle to detect and estimate the multipath/NLoS effects using leftover terms. This research assumes that at least two satellites are free from the impact of multipath/NLoS effects, which can cover most conditions according to the previous study [23]. Under this assumption, $L^{s}\left(t\right)$ without multipath/NLoS effects is expected to follow the distribution $N\left(c\;\cdot \;dt_{\mathrm{rcv}},\sigma^{2}\right)$, forming a cluster whose size is controlled by $e_{r}^{s}\left(t\right)$. On the other hand, $L^{s}\left(t\right)$ with multipath/NLoS effects should follow the distribution $N\left(c\;\cdot \;dt_{\mathrm{rcv}}+F^{s}\left(t\right),\sigma_{F}^{2}\right)$, exhibiting a mean $F^{s}\left(t\right)$. Given that $F^{s}\left(t\right)$ is significantly larger than both σ and $\sigma_{F}$, the leftover terms affected by multipath/NLoS will be separated from the cluster formed by leftover terms without multipath/NLoS effects. However, it is unlikely that the leftover terms impacted by multipath/NLoS can form a consistent cluster due to the distinct values of $F^{s}\left(t\right)$ for each satellite resulting from different reflection paths.

Based on the previous analysis, clustering algorithms can be utilized to identify the largest cluster without multipath/NLoS, subsequently enabling the determination of leftover terms affected by multipath/NLoS.

If the assumption is that at least two satellites are free from multipath/NLoS effects, the method will be unable to form a cluster consisting of $L^{s}\left(t\right)$ without multipath/NLoS. Consequently, the multipath/NLoS estimation method will declare a failure rather than providing an inaccurate estimate. Additionally, the clustering method may make it difficult to identify small-value $F^{s}\left(t\right)$ because the quantities are close to $e_{r}^{s}\left(t\right)$. However, considering the fact that, as previously recalled, $e_{r}^{s}\left(t\right)$ is expected to range in the order of about ±1 m, those $F^{s}\left(t\right)$ values close to $e_{r}^{s}\left(t\right)$ will only produce minor impact positioning errors due to their small values. As a result, the main focus of this research is on those sufficiently large values of $F^{s}\left(t\right)$ that will induce detrimental loss of accuracy in the navigation solutions.

### 3.2. DBSCAN, a Clustering Algorithm for Multipath/NLoS Estimation

After having motivated the use of a clustering algorithm as a means to distinguish the presence of multipath/NLOS effects, this section will introduce the specific clustering algorithm, DBSCAN, employed in this work, discussing the parameter selection that makes it suitable to accomplish the task.

DBSCAN is a minimum density level estimation that clusters data based on the density. This algorithm first specifies two parameters:

1. minPts: the minimum number of points to form a cluster.
2. ε: the maximum distance between two points to consider them neighbors.

Then, every data point will be classified into three types:

1. **Core points**: the data points can find at least minPts neighbors within the radius ε.
2. **Non-core points (border points)**: within radius ε, the data points can find at least one core point but have no more than minPts neighbors.
3. **Outliers**: the data points do not satisfy either the definition of core points or the one of non-core points.

**Figure 1:**
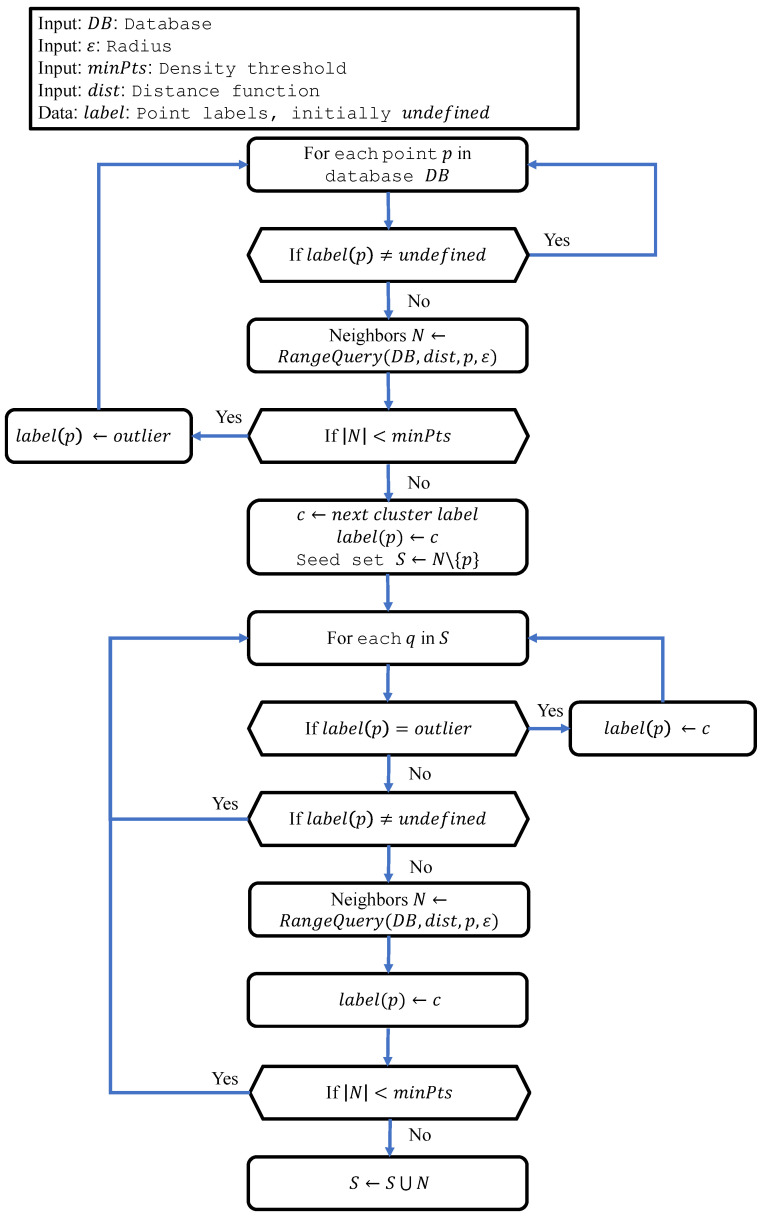
Flowchart of the DBSCAN algorithm.

DBSCAN can be described using the flowchart in Figure 1. In the first step of DBSCAN, the RangeQuery function is employed to identify all the neighbors of a specific point. This function finds all the data points in DB whose distance to the current point *p* is closer than ε. Here, dist is a function used to compute the distance between two data points.

Upon identifying a core point, all its neighbors are assigned to the same cluster as that core point. If any of these neighbors is itself a core point, the neighbors of this new core point are classified into the same cluster. This process is iterated until all data points are clustered. Points that do not satisfy the aforementioned conditions are designated as outliers.

DBSCAN is a suitable clustering solution to address the multipath/NLoS problem since the parameter minPts establishes the minimum number of leftover terms $L^{s}\left(t\right)$ needed to confirm that they belong to the distribution $N\left(c\;\cdot \;dt_{\mathrm{rcv}},\sigma^{2}\right)$. Meanwhile, the receiver noise $e_{r}^{s}\left(t\right)$ acts as a reference for setting the parameter ε, thus controlling the boundary of the cluster free from multipath/NLoS effects.

### 3.3. Implementation of Multipath/NLoS Estimation Based on DBSCAN

In total, four steps are involved in estimating the $F^{s}\left(t\right)$ term. The fundamental concept is to isolate $c\;\cdot \;dt_{\mathrm{rcv}}$ from $L^{s}\left(t\right)$ for the pseudorange containing multipath/NLoS using the DBSCAN algorithm.

In the initial step, all *M* leftover terms according to (2) for a certain epoch are computed:

(5) $$L^{s}\left(t\right),s=1,\ldots ,M$$

The second step is detecting which leftover terms are affected by multipath/NLoS. By setting the parameters ε, minPts, and dist, DBSCAN will analyze and identify clusters that meet the specified parameter configuration. Let ${\tilde{L}}^{s}\left(t\right)$ be the leftover terms belonging to the largest cluster assumed to represent the one free from multipath/NLoS effects. The leftover terms not belonging to the largest cluster are determined to be affected by multipath/NLoS effects.

Although such a condition is very rare, sometimes $L^{s}\left(t\right)$ values with multipath/NLoS effects from different satellites could be similar and, therefore, form a cluster. Considering the low probability of different multipath/NLoS biases being similar to each other, selecting the largest cluster further decreases the risk of incorrectly judging the multipath/NLoS-free cluster.

The third step is to estimate $dt_{\mathrm{rcv}}$ by computing the mean value of ${\tilde{L}}^{s}\left(t\right)$:

(6) $${\hat{dt}}_{\mathrm{rcv}}=mean\left({\tilde{L}}^{s}\left(t\right)\right)$$

The final step is to estimate the biases on pesudorange measurements due to multipath/NLoS effects. According to (3), this estimation is completed by separating $c\;\cdot \;{\hat{dt}}_{\mathrm{rcv}}$ from $L^{s}\left(t\right)$ using the estimated ${\hat{dt}}_{\mathrm{rcv}}$ obtained from the previous step.

(7) $${\hat{F}}^{s}\left(t\right)=L^{s}\left(t\right)-c\;\cdot \;{\hat{dt}}_{\mathrm{rcv}},\forall s\;\mathrm{out}\;\mathrm{of}\;\mathrm{the}\;\mathrm{largest}\;\mathrm{cluster}$$

## 4. Experiments

### 4.1. Static Experiment

#### 4.1.1. Experimental Setup

**Figure 2:**
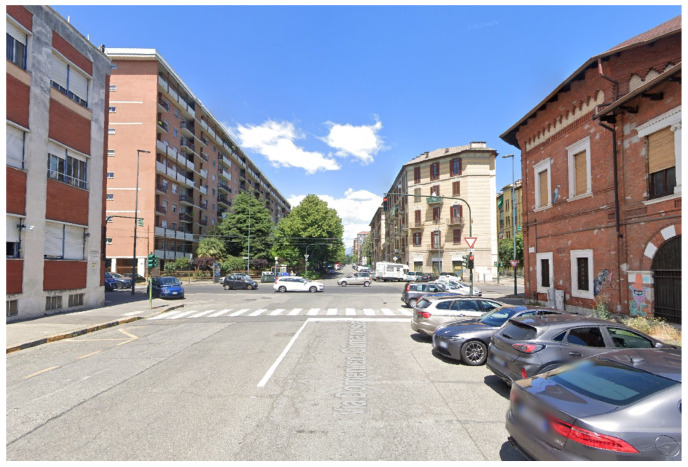
Surrounding buildings of data collection scenario.

**Figure 3:**
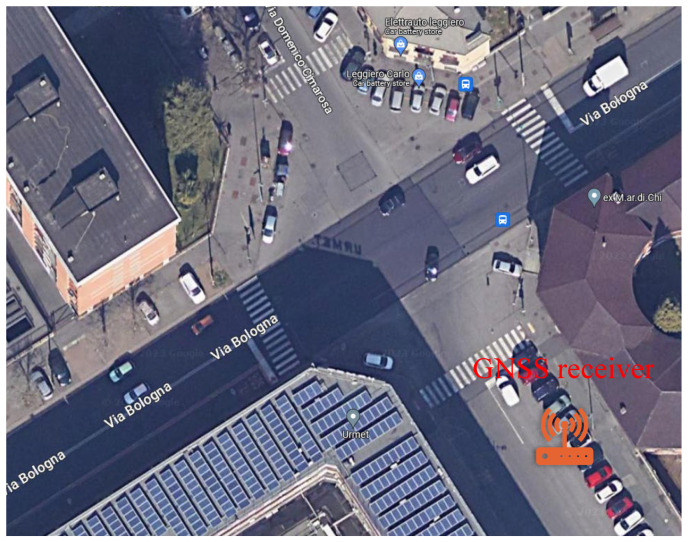
Location of GNSS antenna.

**Figure 4:**
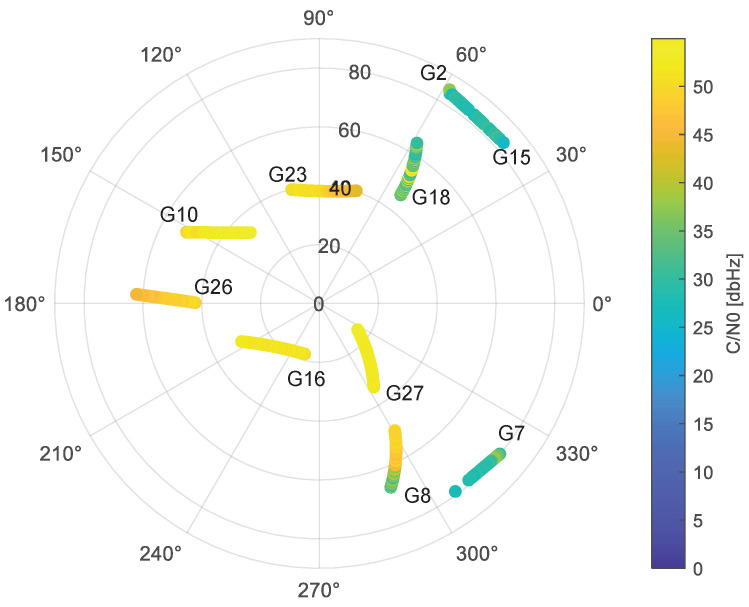
Skyplot with corresponding signal $C/N_{0}$.

A real-world experiment was carried out in a multipath scenario at the Metropolitan City of Turin (Italy), as shown in Figure 2, to demonstrate the proposed method. A Leica GS18 receiver gathered raw GNSS measurements of GPS L1 C/A signals at a rate of 10 Hz at the position depicted in Figure 3. The antenna was fixed and static for the entire collection process. Its position was georeferenced by means of an RTK solution, thus providing the position ground truth for the test. It is important to highlight that, in order to ensure the reliability of the ground truth, only the fixed RTK solutions were chosen and averaged to derive the ground truth under the conditions affected by multipath/NLoS. The GNSS data collection lasted for around 40 min. The skyplot with carrier-power-to-noise-density ratio ($C/N_{0}$) is provided in Figure 4. The receiver was deployed close to the buildings on the east side of the road but kept a short distance away from the buildings on the west side.

To obtain the $L^{s}\left(t\right)$ values of the pseudorange measurements, all the corrections and models were computed according to Table 1. As far as the DBSCAN is concerned, the minPts and ε parameters were set as 2 and 2 m, respectively. Given the limited satellite visibility in urban areas, minPts was set to 2 since at least 2 points are required to confirm membership of the same cluster. For the parameter ε, it should represent the maximum distance between two points within the same cluster. In our specific context, this maximum distance of the distribution $N\left(c\;\cdot \;dt_{\mathrm{rcv}},\sigma^{2}\right)$ is dependent on the variance $\sigma^{2}$, which is associated with receiver noise (±1 m), and it is then set to 2 m.

DB in this research contains all the $L^{s}\left(t\right)$ values, which are one-dimensional data. Hence, dist is set to compute the absolute value of the difference between two $L^{s}\left(t\right)$ values.

#### 4.1.2. Experimental Results of Multipath/NLoS Estimation

**Figure 5:**
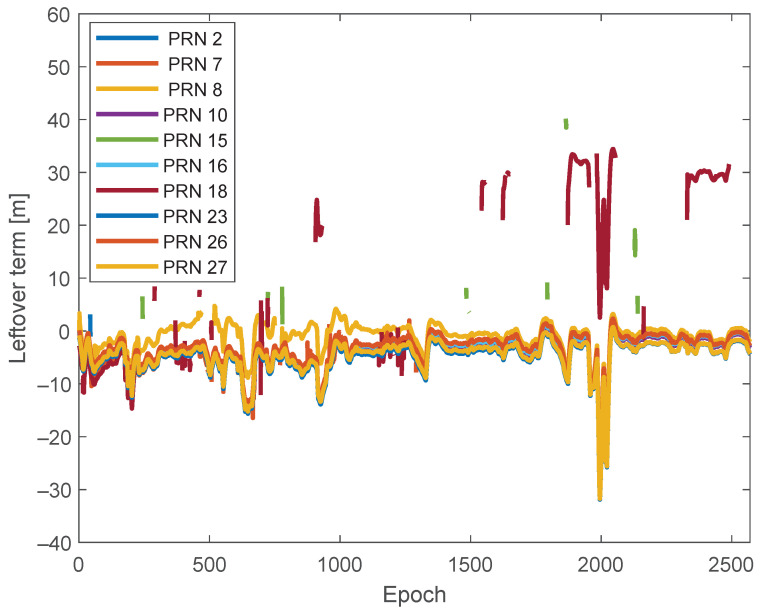
Computed leftover terms $L^{s}\left(t\right)$ for GPS satellites.

As an initial step, all the corrections and models listed in Table 1 were computed using the library provided by RTKLIB [24]. Subsequently, the pseudorange leftover terms $L^{s}\left(t\right)$ for all epochs were calculated applying these corrections and models. Figure 5 depicts all the $L^{s}\left(t\right)$ values, which are marked in different colors to distinguish the corresponding satellite pseudorandom noise. It can be seen that most $L^{s}\left(t\right)$ values exhibit similar trends, indicating they are influenced solely by $c\;\cdot \;dt_{\mathrm{rcv}}$ and $e_{r}^{s}\left(t\right)$. Conversely, certain $L^{s}\left(t\right)$ values deviate from this trend, suggesting the presence of the large biases $F^{s}\left(t\right)$ caused by multipath/NLoS effects. PRN 18 shows significantly different $L^{s}\left(t\right)$ values compared to other PRNs, indicating a high probability of suffering from multipath/NLoS effects. Based on the skyplot in Figure 4 and the surroundings of the GNSS receiver in Figure 3, the reflected signal for PRN 18 may be attributed to the building on the west side of the road.

**Figure 6:**
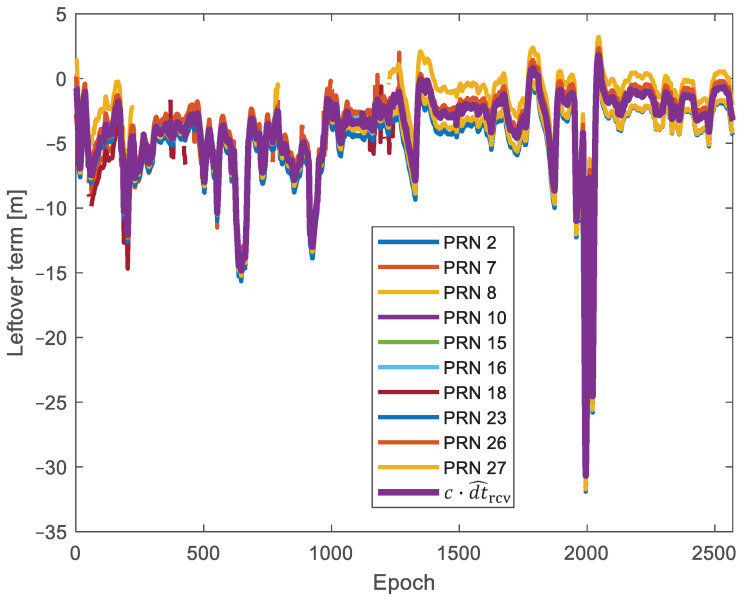
Leftover terms in the largest cluster determined by DBSCAN and $c\;\cdot \;{\hat{dt}}_{\mathrm{rcv}}$.

Then, Figure 6 shows all the ${\tilde{L}}^{s}\left(t\right)$ values in the largest cluster provided by DBSCAN, together with their mean value estimated as $c\;\cdot \;{\hat{dt}}_{\mathrm{rcv}}$.

**Figure 7:**
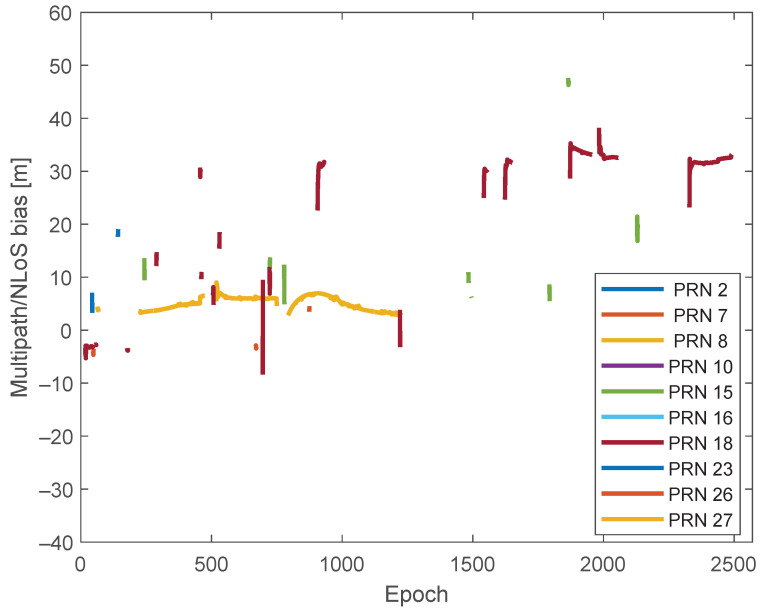
Estimated multipath/NLoS bias on pseudoranges.

Given the estimated $c\;\cdot \;{\hat{dt}}_{\mathrm{rcv}}$, the ${\hat{F}}^{s}\left(t\right)$ values can be obtained for the leftover terms not belonging to the largest cluster based on (7), and they are depicted in Figure 7. It can be seen that positive values of ${\hat{F}}^{s}\left(t\right)$ are observed more frequently than negative values, consistent with the theoretical analysis in [2]. In fact, multipath can introduce both positive and negative biases to pseudoranges, while NLoS tends to produce only positive biases. Therefore, positive biases are more frequent.

After obtaining the ${\hat{F}}^{s}\left(t\right)$, it is necessary to prove the reliability of these estimates. Since there are no ground truth values for pseudorange measurement, in this work, we use a Generalized Least Squares (GLSs) positioning algorithm operating on pseudoranges corrected for the estimated values of ${\hat{F}}^{s}\left(t\right)$. Even if, as explained in the experimental setup, a reliable ground truth for the position can be obtained, it is not straightforward to obtain the ground truth for each pseudorange measurement since, in particular, a true value for the receiver clock ${dt}_{r}\left(t\right)$ in (1) is not known. Only the estimated value is available, and such estimates are affected by the presence of multipath/NLoS biases. For this reason, to validate the accuracy of the estimated multipath/NLoS biases, we prove the effectiveness of the method at the Position, Velocity, and Time level by comparing the accuracy of the solution obtained using pseudoranges with and without the application of the correction for the multipath/NLoS biases. Following this principle, new positioning results are computed using a new GNSS dataset, which is generated by compensating for ${\hat{F}}^{s}\left(t\right)$ in the pseudorange measurements. This compensating action is conducted by subtracting the estimated ${\hat{F}}^{s}\left(t\right)$ from the corresponding pseudorange measurements.

**Figure 8:**
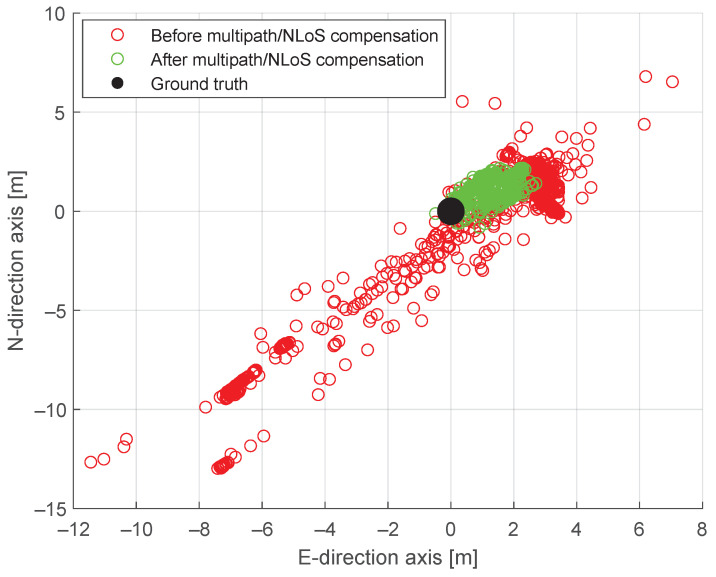
Positioning scatters before and after pseudorange multipath/NLoS compensation in the horizontal plane.

**Figure 9:**
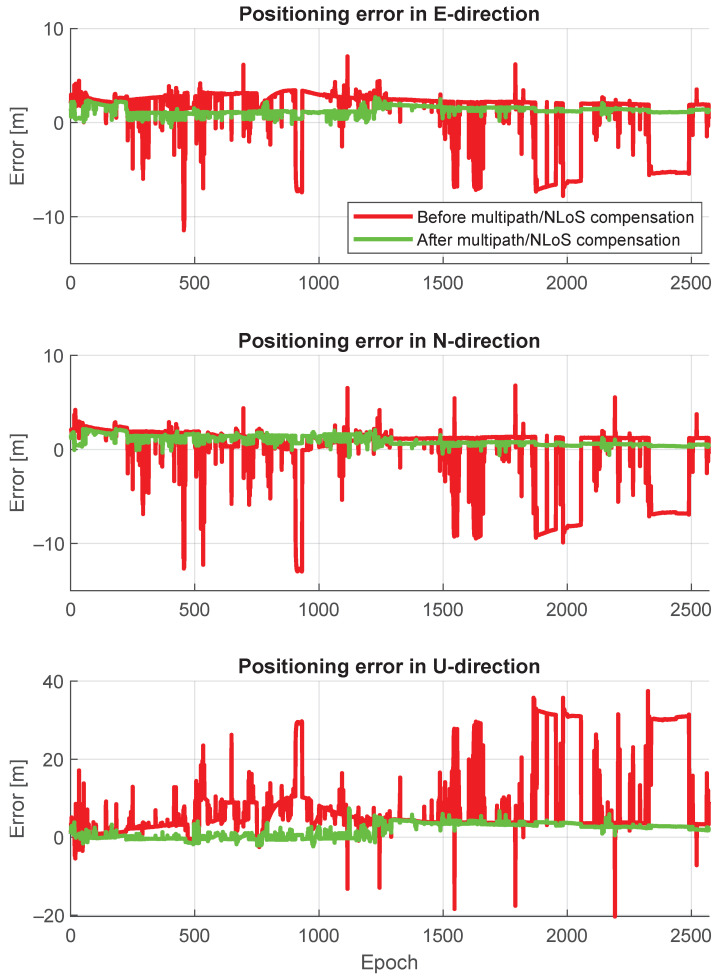
Positioning error time series before and after pseudorange multipath/NLoS compensation in East, North, and Up directions.

Figure 8 compares positioning scatters with and without multipath/NLoS compensation. Given that ${\hat{F}}^{s}\left(t\right)$ is estimated from the leftover term, which contains ground truth information, the scatter of positioning after multipath/NLoS compensation is concentrated around the true position with high accuracy. This indicates that the computed ${\hat{F}}^{s}\left(t\right)$ values closely approximate the actual pseudorange biases caused by multipath/NLoS effects. Figure 9 shows the positioning error time series in both the horizontal and vertical directions before and after the multipath/NLoS compensation. It is observed that the positioning errors are smaller in magnitude than the estimated ${\hat{F}}^{s}\left(t\right)$ for the dataset without multipath/NLoS compensation. This occurs because the locations are computed based on both the pseudorange measurements with and without multipath/NLoS effects. The pseudorange measurements without multipath can provide accurate positioning information, thereby mitigating the multipath/NLoS positioning accuracy degradation.

**Figure 10:**
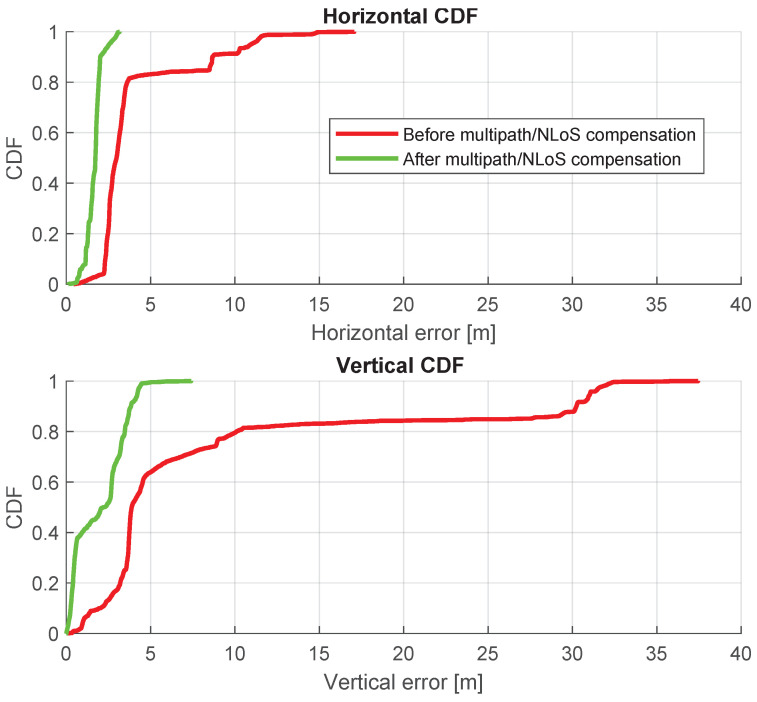
CDFs of positioning errors before and after pseudorange multipath/NLoS compensation in horizontal and vertical directions.

The Cumulative Distribution Functions (CDFs) of these positioning errors are provided in Figure 10. The CDF illustrates the distribution of positioning errors, serving a crucial role in assessing the enhanced positioning accuracy after multipath/NLoS compensation. The 3D Root Mean Squared Error (RMSE) can be improved from 13.92 m to 3.01 m (reduced by 78%) using the new dataset that compensates for multipath/NLoS biases.

Table 2 presents the horizontal and vertical errors (at the 95th percentile of the CDFs) derived from the GNSS datasets both before and after the multipath/NLoS compensation. The positioning solutions after the multipath/NLoS compensation in Figure 8 still contain slight biases. However, these positioning results meet the standard accuracy performance criteria for GPS, as outlined in [25].

### 4.2. Dynamic Experiment

#### 4.2.1. Experimental Setup

**Figure 11:**
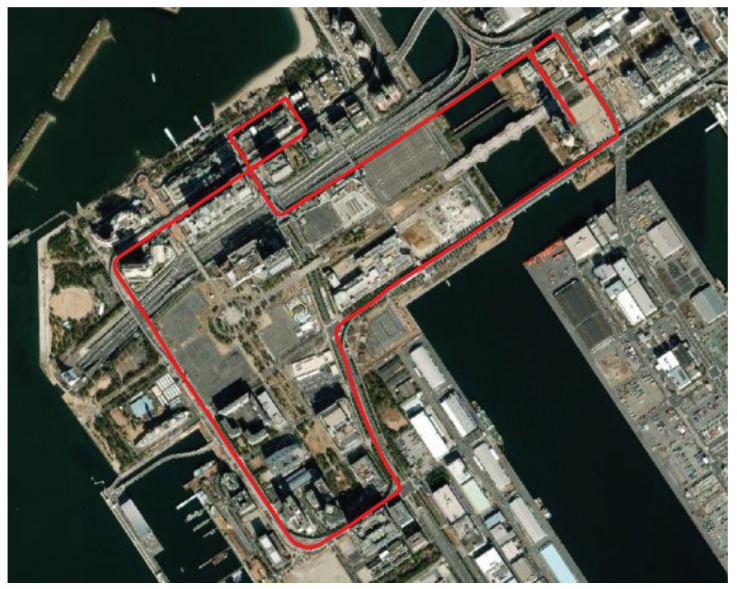
Vehicle trajectory for the Odaiba dataset [27].

The dynamic experiment utilizes the UrbanNav dataset provided by Hong Kong Polytechnic University [26]. The specific dataset used, named Odaiba, was collected within the challenging urban canyons of Tokyo. This experiment focuses on single-frequency GPS L1 C/A signals. The GNSS data were acquired at a rate of 10 Hz using Trimble NetR9. The ground truth information was provided by the integration of RTK and INS, offering an RMSE of 5 cm and a frequency of 10 Hz under the multipath/NLoS conditions. The trajectory for the entire experiment is illustrated in Figure 11. It is important to note that the experiment was carried out based on a segment of the entire trajectory featuring strong multipath/NLoS interference.

The settings for DBSCAN totally inherit the parameters provided in Section 4.1.1.

#### 4.2.2. Experimental Results of Multipath/NLoS Estimation

**Figure 12:**
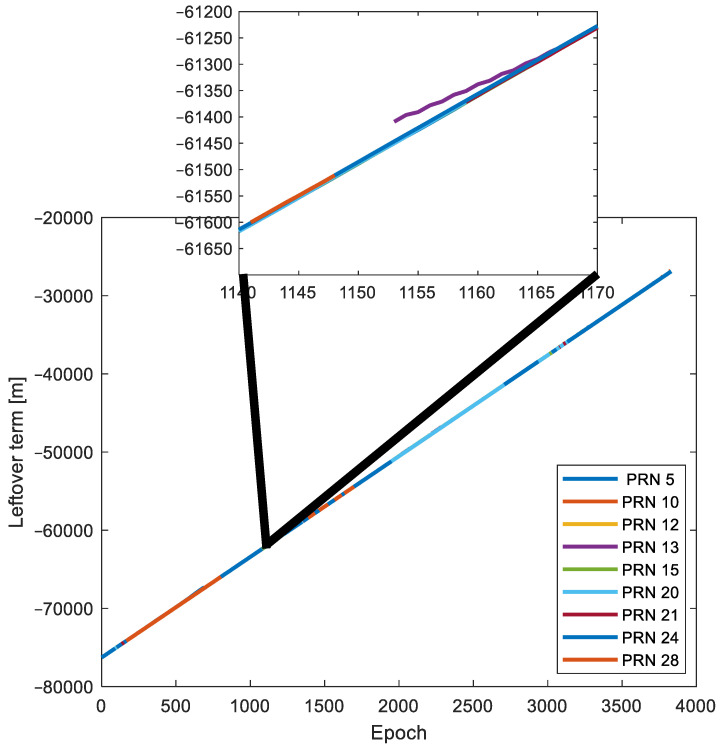
Computed leftover terms $L^{s}\left(t\right)$ for GPS satellites.

Figure 12 illustrates all the $L^{s}\left(t\right)$ values for the pseudorange measurements computed using the corrections and models outlined in Table 1. It is worth noting that, due to the Trimble receiver’s relatively large clock bias and drift, the dominating factor in the $L^{s}\left(t\right)$ values is the term $c\;\cdot \;dt_{\mathrm{rcv}}$. Consequently, the visual impact of $L^{s}\left(t\right)$ values influenced by multipath/NLoS effects may not be immediately apparent in Figure 12. However, the DBSCAN algorithm is still able to recognize and segregate $L^{s}\left(t\right)$ values affected by multipath/NLoS effects. This is achieved by identifying deviations from the general trend formed by $L^{s}\left(t\right)$ values that are free from multipath/NLoS effects. This can be observed in the magnified section of Figure 12.

**Figure 13:**
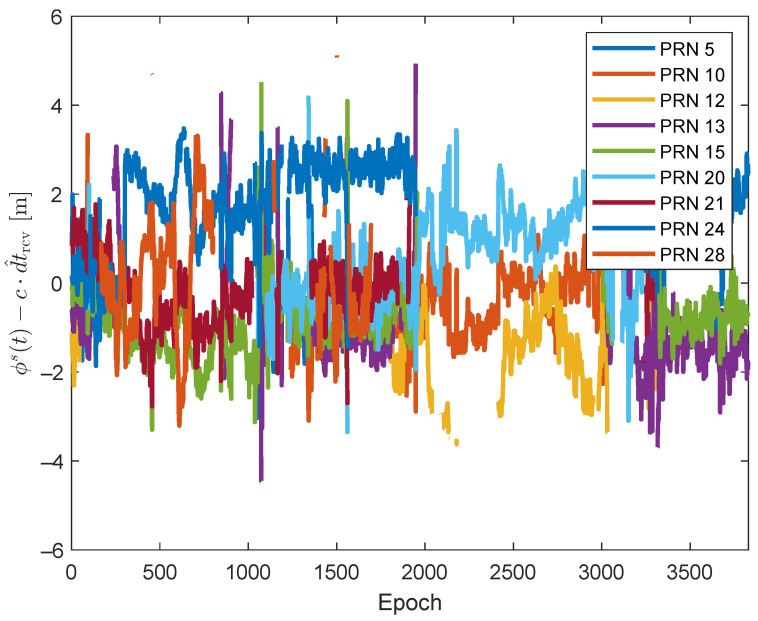
$L^{s}\left(t\right)-c\;\cdot \;{\hat{dt}}_{\mathrm{rcv}}$ in the largest cluster determined by DBSCAN.

To mitigate the impact of clock bias and drift on visualization, the DBSCAN algorithm is initially employed to identify $L^{s}\left(t\right)$ values unaffected by multipath/NLoS effects within the largest cluster. Subsequently, the time series of the estimated $c\;\cdot \;{\hat{dt}}_{\mathrm{rcv}}$ is computed by averaging all the $L^{s}\left(t\right)$ values within this cluster for each epoch. Figure 13 illustrates the time series of $L^{s}\left(t\right)-c\;\cdot \;{\hat{dt}}_{\mathrm{rcv}}$ within the largest cluster. As per (3), $L^{s}\left(t\right)-c\;\cdot \;{\hat{dt}}_{\mathrm{rcv}}$ should only be contributed by receiver noises for the cluster free from multipath/NLoS effects. The majority of the values in Figure 13 fall within the ±2 m range, which has a similar level to the receiver noise. This observation validates the analysis in Section 3.1, which further shows the effectiveness of the DBSCAN algorithm.

**Figure 14:**
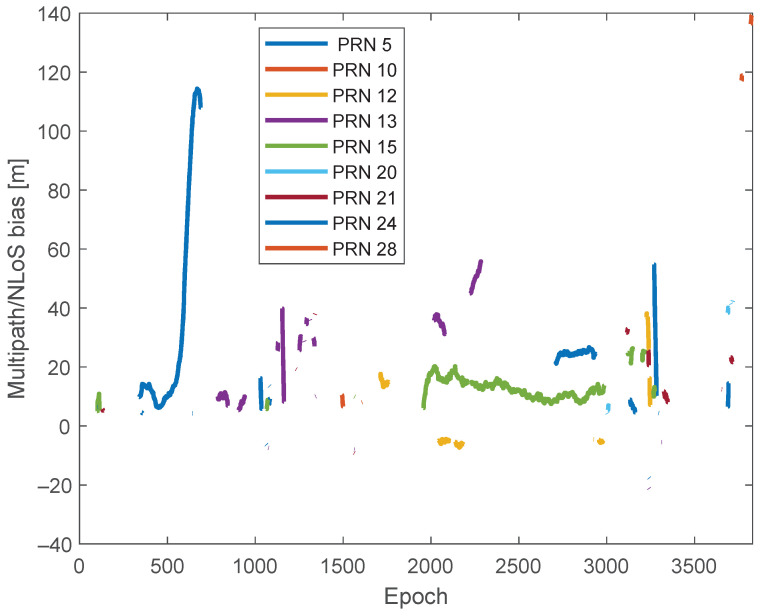
Estimated multipath/NLoS bias on pseudorange.

Then, the ${\hat{F}}^{s}\left(t\right)$ values can be separated from the leftover terms out of the largest cluster based on (7). Figure 14 presents ${\hat{F}}^{s}\left(t\right)$ for each satellite. It is observed that PRN 5 produced significant multipath/NLoS biases, reaching up to 120 m. This is more likely caused by NLoS rather than multipath, considering that the maximum multipath bias for GPS L1 C/A signals is estimated to be around 70 m, as discussed in the theoretical analysis in [18].

Like the static dataset, a new GNSS dataset is generated by applying these ${\hat{F}}^{s}\left(t\right)$ to pseudorange measurements to evaluate the effectiveness of the estimated ${\hat{F}}^{s}\left(t\right)$ values. Subsequently, new positioning results are obtained using these adjusted pseudoranges.

**Figure 15:**
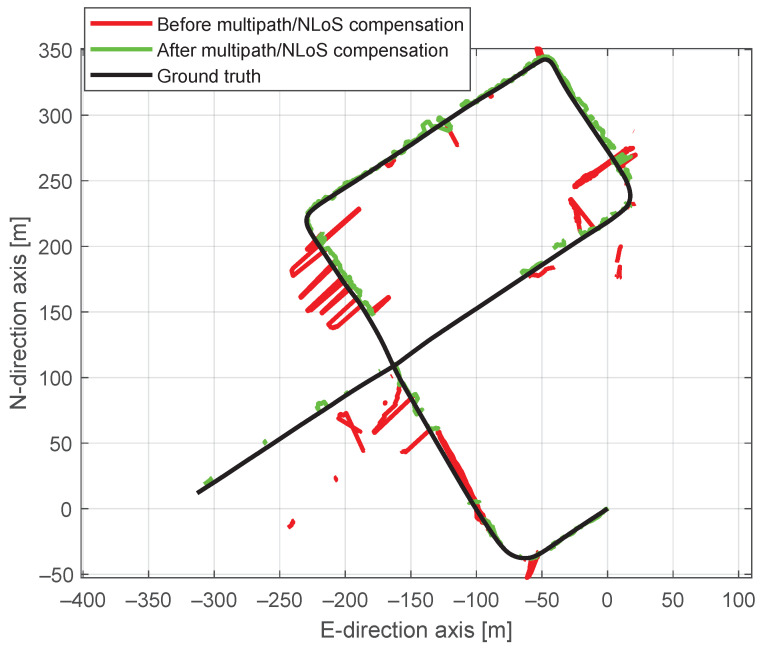
Positioning trajectory before and after pseudorange multipath/NLoS compensation in the horizontal plane.

**Figure 16:**
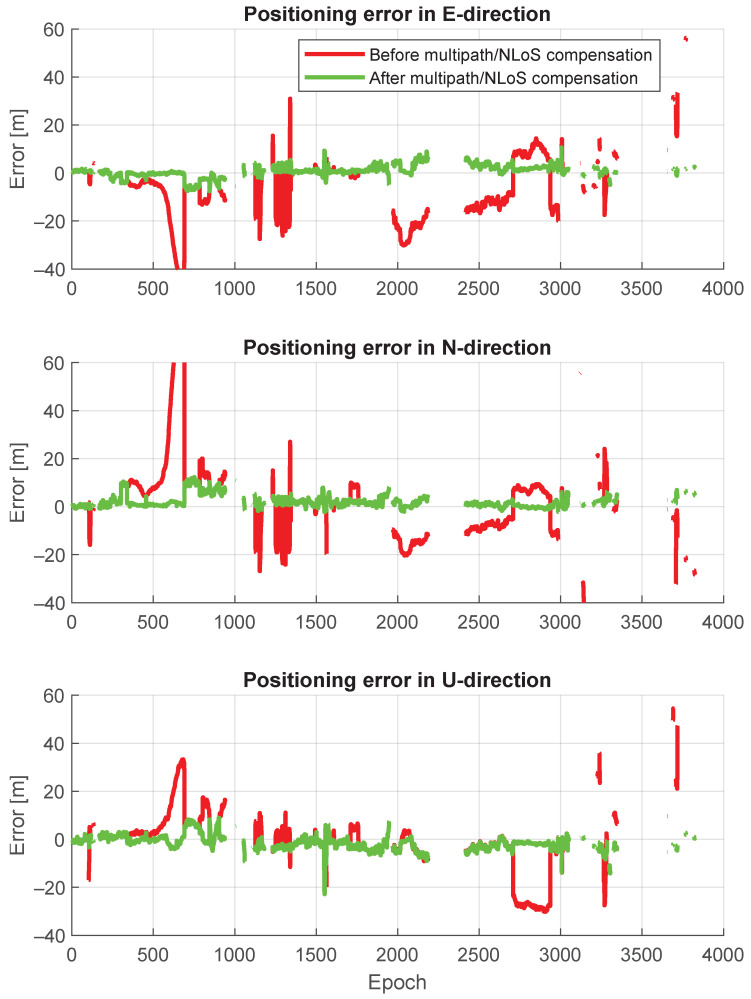
Positioning error time series before and after pseudorange multipath/NLoS compensation in East, North, and Up directions.

**Figure 17:**
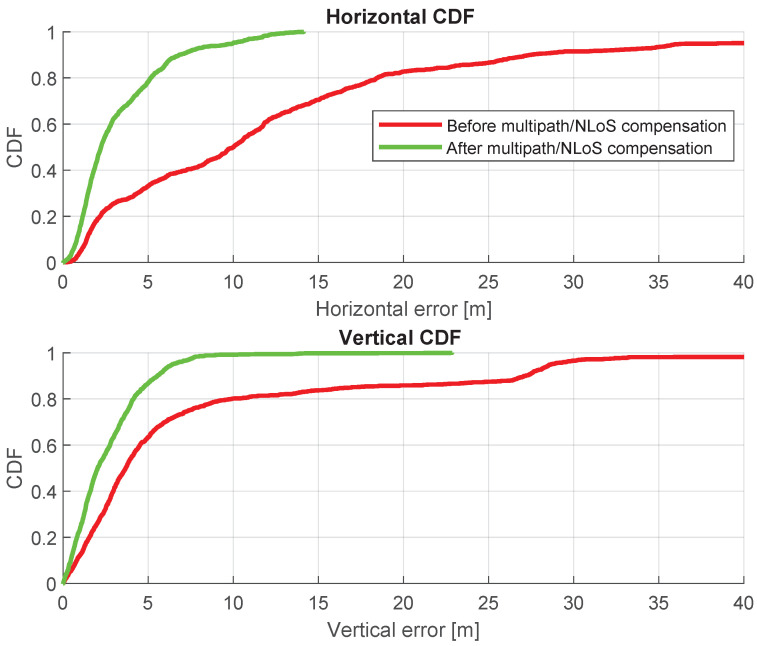
CDFs of positioning errors before and after pseudorange multipath/NLoS compensation in horizontal and vertical directions.

Figure 15 shows a comparison of positioning trajectories with and without multipath/NLoS compensation. The positioning accuracy after the multipath/NLoS compensation proves that these computed ${\hat{F}}^{s}\left(t\right)$ values are close to the real pseudorange biases caused by multipath/NLoS effects. Figure 16 illustrates the time series of positioning errors in both the horizontal and vertical directions before and after the multipath/NLoS compensation. The CDFs of these positioning errors are presented in Figure 17. The 3D RMSE shows a significant improvement, decreasing from 26.0103 m to 5.5094 m (reduced by 79%), using the new dataset that compensates for multipath/NLoS biases.

Table 3 provides the horizontal and vertical errors, specifically the 95th percentile of the CDFs, obtained from the GNSS datasets before and after the compensation for multipath/NLoS effects.

## 5. Conclusions

This study proposed a post-processing method to calculate the pseudorange biases resulting from multipath/NLoS effects. Leveraging the physical models and corrections from IGS, pseudorange leftover terms were computed. The application of the DBSCAN algorithm facilitated the separation of the receiver clock parameters.

The novelty of this paper can be summarized in three key aspects. Firstly, the paper defined a novel leftover term derived from insights into the pseudorange equation. Secondly, through theoretical analysis, two probability models for this leftover term were established under conditions with and without multipath/NLoS effects. The analysis demonstrates that leftover terms without multipath/NLoS effects can form a cluster. Finally, the paper proposed a post-processing procedure based on a clustering algorithm for estimating multipath/NLoS biases.

The experimental results, derived from two real-world datasets in challenging GNSS scenarios for both static and dynamic conditions, demonstrate the effectiveness of the proposed method. Firstly, the successful estimation of receiver clock parameters ${\hat{dt}}_{\mathrm{rcv}}$ was achieved using the DBSCAN method. Secondly, the pseudorange biases due to multipath/NLoS effects were isolated from the leftover terms. Finally, compensating for the estimated pseudorange biases in the measurement results in a significant improvement in 3D positioning accuracy. The 3D positioning RMSE was reduced by 78% (from 13.9217 m to 3.0121 m) and by 79% (from 26.0103 m to 5.5094 m) for the static dataset and dynamic dataset, respectively. The experimental results highlight the effectiveness of the proposed method.

While the proposed method has demonstrated promising results, as outlined earlier, its application is subject to certain limitations. For instance, in scenarios where the interference leading to multipath/NLoS effects is particularly strong, biases may affect all the measurements. Under such extreme conditions, the clustering algorithm may fail as there may be insufficient measurements unaffected by the influence of multipath/NLoS effects.

In the future, several aspects will be considered for extending the proposed multipath/NLoS estimation method, including its application to multi-constellation GNSS systems. Additionally, it would be valuable to explore the factors that influence the accuracy of the clustering algorithms employed in this context.

## Figures and Tables

**Table 1:** Corrections and models for pseudorange measurement.

Term	Computation Method
$p_{r}^{s}\left(t\right)$	Measurements from the receiver
$r^{s}\left(t\right)$	IGS final precise orbits products
$r_{r}\left(t\right)$	RTK or RTK/INS positioning solutions
$\xi_{r}^{s}\left(t\right)$	Absolute IGS phase center corrections (igs14.atx)
$I^{s}\left(t\right)$	Final solution of IGS combined GIMs
$T^{s}\left(t\right)$	Saastamoinen model
$d^{s}$	TGD provided by navigation messages
$dt^{s}\left(t\right)$	Clock biases of the satellites from navigation messages
$\delta t_{\mathrm{stc}}^{s,\mathrm{rel}}\left(t\right)$	See Appendix A
$\delta t_{\mathrm{clk}}^{s,\mathrm{rel}}\left(t\right)$	See Appendix B

**Table 2:** Horizontal and vertical positioning errors at 95th percentile of the CDFs.

	Horizontal [m]	Vertical [m]
Before Compensation	10.94	31.04
After Compensation	2.54	4.22

**Table 3:** Horizontal and vertical positioning errors at 95th percentile of the CDFs.

	Horizontal [m]	Vertical [m]
Before Compensation	38.75	28.68
After Compensation	9.98	6.43

## Data Availability

The static GNSS data used in this research are available from Yihan Guo (yihan.guo@polito.it) upon reasonable request; The dynamic GNSS data can be downloaded from https://github.com/weisongwen/UrbanNavDataset (accessed on 25 October 2023).

## Appendix A. Shapiro Effect Correction for $\delta t_{\mathrm{stc}}^{s,\mathrm{rel}}(t)$

Earth’s gravitational field causes the Shapiro effect, which results in a time delay for GNSS signal propagation. The correction for the Shapiro effect can be compensated by

(A1) $$\delta t_{\mathrm{stc}}^{s,\mathrm{rel}}=\frac{2\mu }{c^{3}}\ln\left(\frac{∥r^{s}∥+∥r_{r}∥+\rho_{r}^{s}}{∥r^{s}∥+∥r_{r}∥-\rho_{r}^{s}}\right)$$

where

- μ is the gravitational constant of Earth.

## Appendix B. Relativistic Clock Correction for $\delta t_{\mathrm{clk}}^{s,\mathrm{rel}}(t)$

The relativity affects the satellite clock through both the satellite motion and the unsteady gravitation field. Although the oscillator frequency of the satellite clock has been intentionally offset to compensate for the relativistic effect, the elliptical satellite orbits cause deviations to the set offset. The compensation due to the orbit eccentricity can be calculated from

(A2) $$\delta t_{\mathrm{clk}}^{s,\mathrm{rel}}=-\frac{2}{c^{2}}\sqrt{a\mu }e\sin E$$

- *a* is the orbit semimajor axis;
- *e* is the orbit eccentricity;
- *E* is the eccentric anomaly of the satellite.
